# A Study on the Visibility and Radiological Characteristics of a Normal Appendix in Patients Undergoing Non-contrast Computed Tomography at Teaching Hospital Jaffna, Sri Lanka

**DOI:** 10.7759/cureus.102357

**Published:** 2026-01-26

**Authors:** Sreekanthan Gobishangar, Pushparatnam Abiharan, Anton Jenil, Sivagnanaselvam Yohini, Shathana Paramanathan

**Affiliations:** 1 Surgery, Faculty of Medicine, University of Jaffna, Jaffna, LKA; 2 University Surgical Unit, Teaching Hospital Jaffna, Jaffna, LKA; 3 Radiology, Base Hospital Point Pedro, Point Pedro, LKA; 4 Neurological Surgery, Teaching Hospital Jaffna, Jaffna, LKA; 5 Health Sciences, Management and Science University, Kuala Lumpur, MYS

**Keywords:** appendicitis, appendix, computed tomography, mcburney's point, mcburney’s point, normal appendix, retrocaecal, retrocecal

## Abstract

Background

The appendix is a vermiform structure with a variable position, length, and thickness. Imaging plays a critical role in diagnosing an inflamed appendix, with non-contrast computed tomography (NCCT) being an effective modality when ultrasound is inconclusive. This study evaluates the visibility and anatomical characteristics of a normal appendix using NCCT.

Methods

This retrospective descriptive study was conducted in the Radiology Unit of Teaching Hospital Jaffna, in Jaffna, Sri Lanka, from September 2023 to February 2024, following approval from the Institutional Ethical Review Committee of the hospital. Two hundred individuals were evaluated (past appendectomy patients were excluded). IBM SPSS Statistics software, version 26 (IBM Corp., Armonk, NY, USA) was used, with significance assessed at P ≤ 0.05.

Results

Males were 64.5%. The visibility rate was 89%. The most common positions were retrocecal (27.5%), retroileal (22%), and pelvic (21.5%). The mean length was 7.5 cm, with a base diameter of 6.6 mm, a mid-section of 6.2 mm, and a tip of 5.8 mm. Mean wall thickness was 2.6 mm, fecoliths were found in 15% of cases, and the mean distance of the base from McBurney’s point was 3.32 cm.

Conclusion

NCCT can be used to assess the morphology, position, and presence of fecolith in equivocal cases of suspected appendicitis when ultrasound is inconclusive and contrast is contraindicated. The appendicular diameter and wall thickness can be used as evidence of inflammation. McBurney’s point is a reliable anatomical guide during open appendicectomies. The presence of a fecalith itself is not a reliable indicator of appendicitis.

## Introduction

The appendix is a vermiform (worm-shaped) tubular structure arising from the postero-medial aspect of the cecum, which, except for some variations, lies in the right iliac fossa. The general idea is that the base of the appendix, where all three taenia coli converge, is constant, but the position of the tip, length, and external thickness of the appendix are variable from individual to individual. For several decades of surgical practice, the base of the appendix is presumed to lie deep to the McBurney’s point (one-third of the way from the anterior superior iliac spine to the umbilicus). Still, a wide variation was identified in reality, with only

Thirty-five percent of appendix bases were found to lie within 5 cm of it, 50% within 5-10 cm, and 15% more than 10 cm [1]. The mean length of it is 91.2 mm in males and 80.3 mm in females [2]. The anatomical positions of the appendix were found as follows: pelvic in 55.8%, subcecal in 19%, retroileal in 12.5%, retrocecal in 7%, ectopic in 4.2%, and preileal in 1.5% of the bodies, respectively [2].

The acute inflammation of the appendix (acute appendicitis) is the most common cause of abdominal pain, which commonly requires surgical intervention. The estimated lifetime prevalence of appendicitis is about 7% [3-5]. The reported mortality is <1% in young patients and around 5% in the elderly [6]. A typical presentation is the sudden onset of periumbilical pain, which shifts to the right iliac fossa, accompanied by tenderness and rebound tenderness. As mentioned previously, the variation in the position of the tip is one of the reasons for the variable presentation of appendicitis. A typical presentation occurs only in 50% to 60% of patients [7-8]. The overall diagnostic accuracy clinically, without imaging, approaches 80% with a higher percentage of negative surgeries in females of childbearing age [9].

Imaging, when used in doubtful cases, shortens the time for diagnosis and the time interval to start intervention and reduces complication rates. In Sri Lanka, the modality of choice is an ultrasound scan of the abdomen and pelvis. The overall sensitivity was 55%, and specificity was 95%, with positive and negative predictive values of 81% and 85%, respectively [10]. However, the ultrasound scan is highly operator dependent, and accuracy decreases in obese people. Therefore, an ultrasound scan in strongly suspected cases is not recommended to confirm the diagnosis. Instead, the patient should be filtered for imaging. So imaging has a big role in equivocal cases rather than clinically diagnostic situations [11].

Computed tomography (CT) is one of the primary modalities of choice in diagnosing appendicitis in many well-developed centers. The overall sensitivity and specificity of CT for diagnosing appendicitis were 87.2% and 75.75%, respectively [12]. Administration of IV contrast for enhancement improves the diagnostic accuracy. Traditionally, the diagnosis of acute appendicitis is made by detecting an enlarged appendix (diameter exceeding 6 mm), with wall thickening greater than 3 mm, wall enhancement, and signs of surrounding inflammation such as periappendiceal fat stranding or the presence of free fluid [13].

The overall aim of using imaging as an adjunct is to reduce diagnostic delay, complication rate, and negative appendectomy rate. The unenhanced CT scan, compared to ultrasound, has higher specificity and sensitivity. The ultrasound scan, combined with optional unenhanced helical focused-appendiceal CT, significantly reduces the negative appendectomy rate from 6% to 0% compared with clinical acumen alone [14]. In a study involving 296 patients referred for CT, 123 patients subsequently underwent surgery.

Appendicitis had been correctly predicted in 104 of 108 patients surgically proven to have appendicitis. If no definite inflammatory changes are detected, based on their experience, they recommend that the patient be monitored clinically and that thin-section, unenhanced helical CT is the optimal technique for detecting acute appendicitis in adult patients [8]. The aim of this study was to investigate the visibility rate and anatomical characteristics of the appendix in patients undergoing non-contrast-enhanced computed tomography scans (NCCTs) at Teaching Hospital Jaffna in Jaffna, Sri Lanka. This is a preliminary study that will open a portal to develop radiological criteria for a normal appendix, which can be further evaluated in suspected patients with acute appendicitis to develop standardized criteria for normal vs. inflamed appendix.

## Materials and methods

This study was designed as a retrospective descriptive study. It was conducted in the radiological units of Teaching Hospital Jaffna in Jaffna, Sri Lanka. The study period was from September 2023 to February 2024, following approval from the Ethical Review Committee, Teaching Hospital, Jaffna. The study population consisted of patients who underwent routine NCCT of the kidney, ureter, and bladder (KUB) at the selected hospital for whom appendicitis was not clinically suspected. Two hundred (all patients who underwent NCCT KUB during the study period were included). Patients with a history of appendectomy were excluded, which was verified through phone interviews and the operation theater registry list at Teaching Hospital Jaffna. There were no patients with a past history of appendicitis who were treated conservatively or diagnosed with inflammatory bowel disease who underwent NCCT. More than 50% of the appendix was visualized (satisfactory visualization) in multiplanar reconstruction with zoomed images; a 4 mm slice thickness and standard dose NCCT protocol (soft tissue window) were used in the evaluation. Multiple oblique coronal views are used to measure the length of the appendix. The relationship to McBurney’s point is also analyzed in the oblique coronal view, and McBurney’s point is marked at the skin level for each individual one-third of the way from the anterior superior iliac spine to the umbilicus. The sample size consisted of all patients who underwent NCCT KUB. Statistical analyses were performed using IBM SPSS Statistics software, version 26 (IBM Corp., Armonk, NY, USA). Descriptive statistics included the mean and standard deviation for numerical variables, as well as frequencies and percentages for categorical variables.

## Results

**Figure 1:**
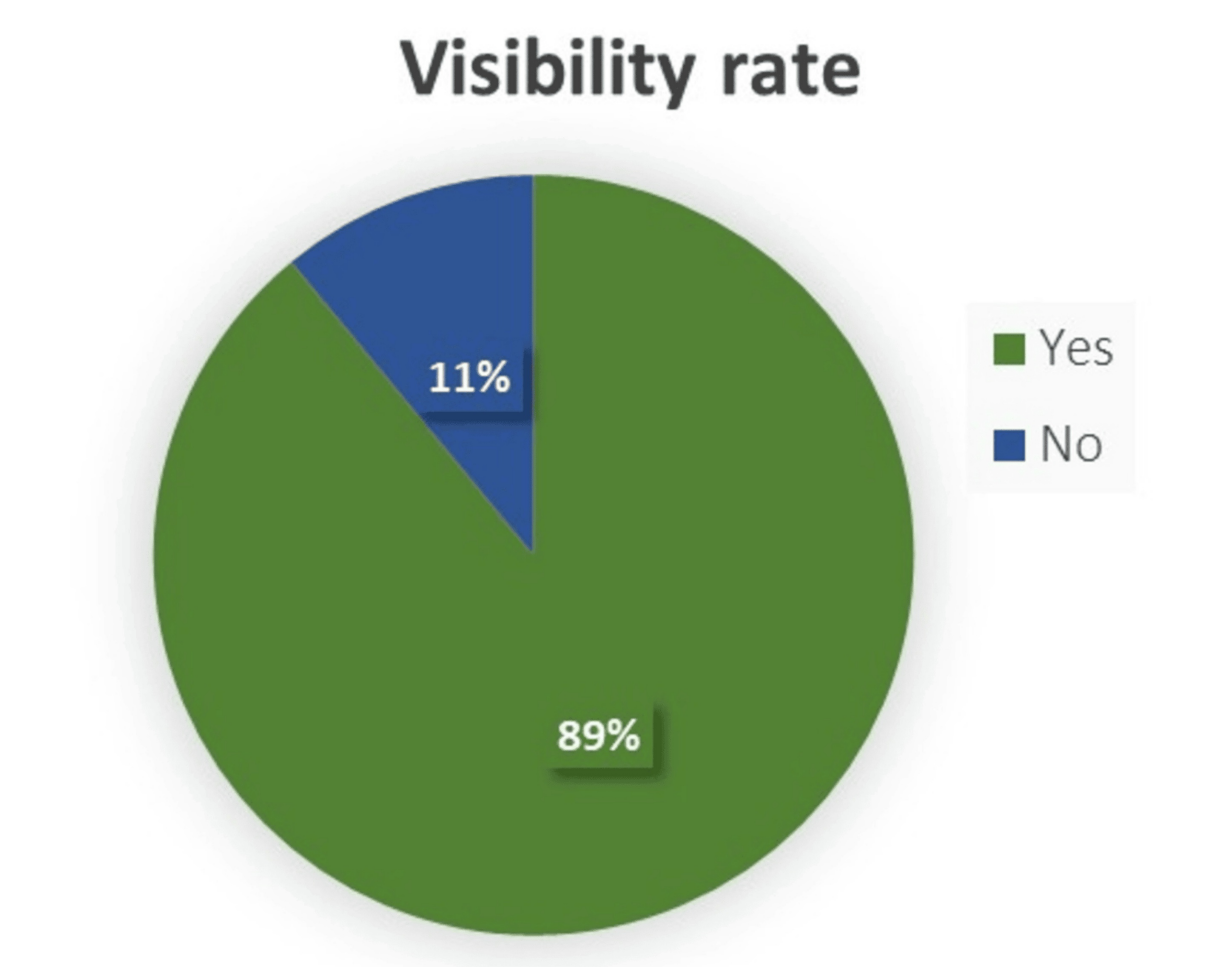
Distribution of rate of visibility of appendix in NCCT NCCT: non-contrast computed tomography

In this study, we evaluated the visibility rates and anatomical characteristics of the normal appendix using NCCT KUB in a sample of 200 individuals who did not exhibit signs of suspected appendicitis. In our study, the males were 64.5%, and the females were 34.5%. The appendix visibility rate was 89% (Figure 1).

**Figure 2:**
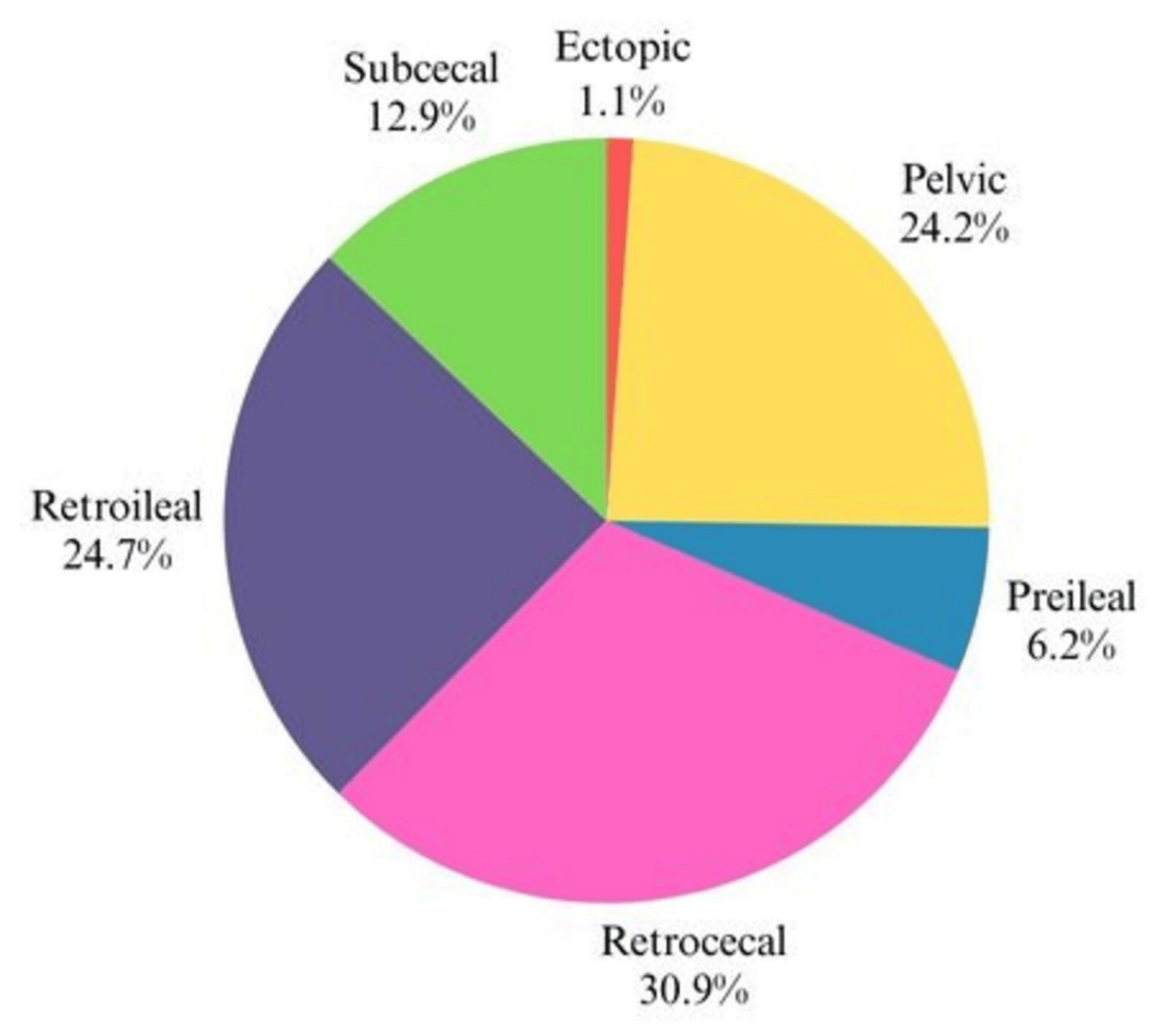
Distribution of the study group based on the anatomical position of the appendix

**Figure 3:**
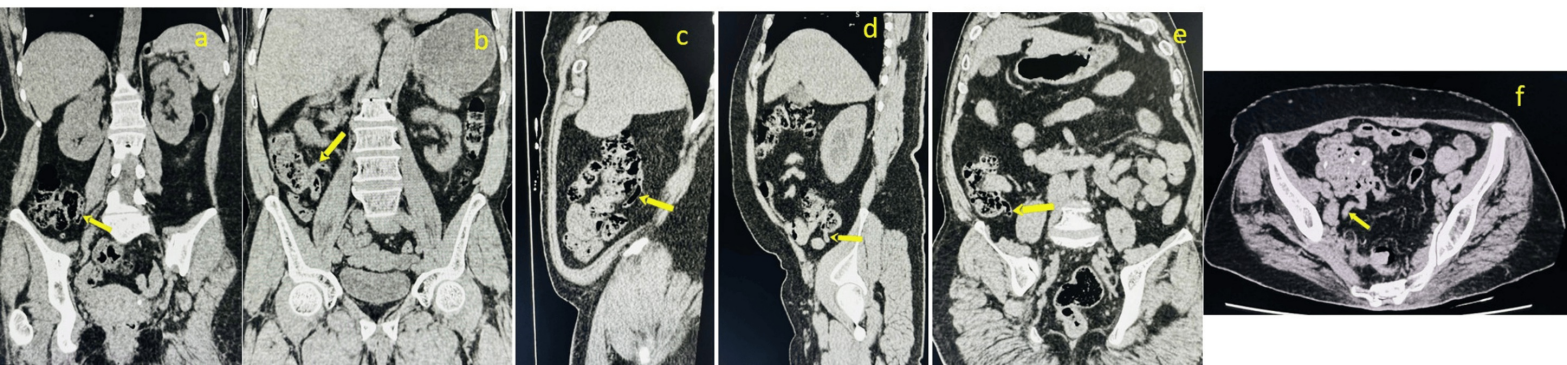
Anatomical positions of the appendix in the NCCT (a) Retroileal position; (b) Preileal position; (c) Retrocaecal position; (d) Subcaecal position; (e) Preileal position; (f) Pelvic position NCCT: non-contrast computed tomography

Notably, no inflamed appendices were identified in this study, further confirming that the sample represents a population without pathological conditions. But fecaliths were present in 15% of cases. The anatomical positioning of the appendix in our study showed distinct differences compared to international studies. Retrocecal positioning was the most common (30.9%), followed by retroileal (24.7%), pelvic (24.2%), subcecal (12.9%), preileal (6.2%), and ectopic (1.1%) (Figures 2, 3).

The mean base position of the appendix was 3.32 cm from McBurney’s point, and the mean apex position was 4.7 cm. The mean thickness of the appendix in our study was 2.6 mm, which aligns with international findings. The mean appendiceal length was 7.5 cm. The mean diameters at the base, mid-section, and tip were 6.6 mm, 6.2 mm, and 5.8 mm, respectively.

## Discussion

Primarily, acute appendicitis is a clinical diagnosis, but with the vast usage of imaging, the negative appendectomy rate is reduced. While an ultrasound scan is the first-line imaging modality, NCCT may have the possibility to be used when ultrasound scan findings are inconclusive, and contrast administration is not feasible. The appendix visibility rate was 89%, which is consistent with international studies reporting an 81% visibility rate [15]. This high visibility rate supports the use of NCCT as an adjunct imaging protocol. Variation in the position of the appendix in our study suggests population-specific anatomical variations. These differences may have clinical implications, particularly for surgeons performing appendectomies or radiologists interpreting imaging in this population. Our study further supports McBurney’s point as a reliable surface landmark during surgical interventions. Our findings support established criteria of a normal appendix on CT, including a two-wall diameter of the appendix <7 mm and a wall thickness <3 mm. These features have been widely reported as key indicators of appendicitis [16].

An appendicolith is defined as hyperattenuating focal material within the lumen of the appendix. While appendicoliths are identified in 15% of cases, Other studies reveal appendicoliths in 0% to 2% of healthy individuals [17-20]. Their association with peri-appendiceal inflammation is a strong indicator of acute appendicitis. However, distinguishing true appendicoliths from bowel content remains challenging in some cases, especially in the absence of contrast. An NCCT may prove to be a valuable clinical tool in equivocal cases where an ultrasound scan is inconclusive, and contrast administration is not feasible. Further studies in large-scale populations, followed by studies using NCCT in patients suspected to have acute appendicitis, may help to develop a standardized criterion to differentiate a normal appendix from an inflamed appendix. We were also able to note that some findings vary from the literature review of NCCT studies, which may be because of population-specific variability or interobserver variability.

Study limitations

Despite these findings, our study has several limitations. Variability in appendix size and morphology among individuals complicates the differentiation between normal and pathological findings.

This highlights the need for further research to establish normative data for the appendix on NCCT. Absence of analyzing factors influencing visibility rate (e.g., patient factors - obesity, abdominal wall thickness, etc. And technical factors such as slice thickness, image quality, etc., are another limitation. There is also no direct comparison between an ultrasound scan and contrast-enhanced CT to estimate the clinical value of NCCT. Finally, our study is limited by its focus on a specific ethnic and geographical population. Broader, multicenter studies involving diverse populations are necessary to confirm these results.

## Conclusions

NCCT can be used as a valuable imaging modality in the assessment of morphology, location, and presence of fecalith. The appendicular diameter >7mm and wall thickness >3mm may serve as radiological evidence of inflammation. The identification of a fecalith can help in decision-making in management, considering the overall clinical picture. McBurney’s point remains a reliable anatomical guide to locate the appendix during open appendectomy. This study can be further followed up with large-scale studies and in patients with suspected appendicitis to determine the utility of NCCT, particularly in cases where ultrasonography yields inconclusive results or when the use of contrast agents is contraindicated. However, variability in appendiceal anatomy and the absence of standardized imaging criteria can pose diagnostic challenges. Therefore, further large-scale, multicenter studies are warranted to establish uniform reference standards for normal appendiceal anatomy vs. an inflamed appendix and to optimize the diagnostic accuracy of NCCT in different clinical settings. Such advancements would contribute to improved diagnostic confidence, reduced negative appendectomy rates, and enhanced patient outcomes where contrast might not be feasible. Furthermore, evaluation using low-dose NCCT as well as limited CT (CT taken in a limited region from McBurney’s point as a center) may further decrease the risk of radiation exposure.
